# Emergent Sources of Prebiotics: Seaweeds and Microalgae

**DOI:** 10.3390/md14020027

**Published:** 2016-01-28

**Authors:** Maria Filomena de Jesus Raposo, Alcina Maria Miranda Bernardo de Morais, Rui Manuel Santos Costa de Morais

**Affiliations:** CBQF—Centro de Biotecnologia e Química Fina—Laboratório Associado, Escola Superior de Biotecnologia, Universidade Católica Portuguesa/Porto, Rua Arquiteto Lobão Vital, Apartado 2511, 4202-401 Porto, Portugal; fraposo@porto.ucp.pt (M.F.J.R.); abmorais@porto.ucp.pt (A.M.M.B.M.)

**Keywords:** seaweeds, algae, microalgae, polysaccharides, PS, prebiotics, microbiota, health benefits, fibre

## Abstract

In recent years, scientists have become aware that human microbiota, in general, and gut microbiota, in particular, play a major role in human health and diseases, such as obesity and diabetes, among others. A large number of evidence has come to light regarding the beneficial effects, either for the host or the gut microbiota, of some foods and food ingredients or biochemical compounds. Among these, the most promising seem to be polysaccharides (PS) or their derivatives, and they include the dietary fibers. Some of these PS can be found in seaweeds and microalgae, some being soluble fibers, such as alginates, fucoidans, carrageenans and exopolysaccharides, that are not fermented, at least not completely, by colonic microbiota. This review gives an overview of the importance of the dietary fibers, as well as the benefits of prebiotics, to human health. The potential of the PS from marine macro- and microalgae to act as prebiotics is discussed, and the different techniques to obtain oligosaccharides from PS are presented. The mechanisms of the benefits of fiber, in general, and the types and benefits of algal fibers in human health are highlighted. The findings of some recent studies that present the potential effects of prebiotics on animal models of algal biomass and their extracts, as well as oligo- and polysaccharides, are presented. In the future, the possibility of using prebiotics to modulate the microbiome, and, consequently, prevent certain human diseases is foreseen.

## 1. Introduction

Recently, there has been a growing understanding that human microbiota, in general, and gut microbiota, in particular, are important. Some diseases might be caused by an imbalance (dysbiosis) among the microorganisms that constitute the microbiota rather than by the presence of a single disease-causing microbe. Examples of diseases associated with microbial dysbiosis include autoimmune and allergic diseases, obesity, inflammatory bowel disease (IBD), and diabetes [1].

In the past two decades, a lot of evidence has come to light regarding the beneficial effects, either for the host or the gut microbiota, of some foods and food ingredients or biochemical compounds. Among these, the most promising seem to be polysaccharides (PS) or their derivatives, namely oligosaccharides or low-molecular-weight (LMW)-PS. These include the so-called dietary fibers.

### 1.1. Definition of Dietary Fiber

Despite the name “fiber”, some of the biochemical compounds of this denomination are not effectively fibers, as they do not possess a fibrous character. They provide similar effects, though.

There are quite different definitions for the term “dietary fiber”. To the Institute of Medicine, a dietary fiber is a “non-digestible carbohydrate (and lignin) naturally found intact in vegetables/plants”. Together with functional fibers, they account for the total fiber content [2]. Functional fibers are fibers obtained from food raw materials or algal biomass through physical, enzymatic or chemical means, and from synthetic carbohydrate polymers. These functional fibers have demonstrated physiological effects that confer health benefits [3].

The American Association of Cereal Chemists (AACC) has a broader concept. For this organization, dietary fiber is “the edible parts of plants or analogous carbohydrates that are resistant to digestion and absorption in the human small intestine with complete or partial fermentation in the large intestine. Dietary fiber includes polysaccharides, oligosaccharides, lignin, and associated plant substances which promote beneficial physiological effects including laxation, and/or blood cholesterol attenuation, and/or blood glucose attenuation” [4].

For the *Codex Alimentarius* Commission, a dietary fiber “means carbohydrate (CHO) polymers with ten or more monomeric units. These CHO polymers may be naturally occurring edible compounds found in the food as consumed, or obtained from food raw material, which are not hydrolyzed by the endogenous enzymes in the small intestine (SI) of humans. Some synthetic CHO polymers are also considered dietary fibres” [5].

However, whatever the definition, some dietary fibers do present additional great prebiotic potential, since they may be fermented by microorganisms at the large intestine level (gut microbiota), and they possess a wide range of physiological benefits for humans. Because they are often understood as practically the equivalent, the concept of prebiotics will be explained later in this review.

### 1.2. Dietary Fibers in Seaweeds

PS can be found in seaweeds and microalgae as (i) cell-wall constituents: cellulose (2%–10% dry weight (DW) in brown algae and 9% DW in *Ulva*), hemicelluloses (9% DW) and lignin (3% DW), in *Ulva*, and some neutral PSs; (ii) storage products: floridean starch (a glucan similar to amylopectin) in red seaweeds, and laminaran in brown macroalgae [6]. In microalgae, PS can also be found as being part of the cell surrounding the glycocalyx and as extracellular polymers (or exopolysaccharides, EPS) (Table 1).

**Table 1:** Dietary fibre content of marine algae in comparison with some foods (vegetables, grains, fruits).

Type of Algae	Total Fiber (% DW)	Soluble Fiber (% DW)	Insoluble Fiber (% DW)	References
**Brown seaweeds**	35–62 ^1^	30–38	5–32	[6]
*Alaria esculenta*	42.86 ^2^			
*Cystoseira abies-marina*	56.34			[7]
*Eisenia bicyclis*	10–75 ^2^			
*Fucus spiralis*	63.88		27–40 ^1^	[7]
*F. vesiculosus* ^a^	50.09 ± 1.77			[8]
	45–59 ^2^			
*Himanthalia elongata* ^a^	32.7	25.7	7.0	[9]
	33–37 ^2^			
*Hizikia fusiforme* ^b^	62.3 ± 0.7			[10]
*Laminaria* sp. ^b^	36.0 ± 5.7		27–40 ^1^	[10]
*L. digitata*	37.3	32.6	4.7	[9]
	36–37 ^2^			
*Laminaria/Saccharina japonica*	10–41 ^2^			
*Saccahrina latissima*	30 ^2^			
*Sargassum fusiforme*	17–69 ^2^			
*Undaria pinnatifida* ^a, b^	35.3–45.9	30.0–33	5.3–6	[9,10,11]
	16–51 ^2^			
**Red seaweeds**				
*Chondrus crispus*	10–34 ^2^			
*Gelidium microdon* ^d^	57.37			[7]
*Gracilaria changii* ^d^	28.0 ^c^			[12]
*Hypnea charoides*	50.3 ± 2.78			[13]
*H. japonica*	53.2 ± 0.56			[13]
*Osmundea pinnatifida*	33.82			[7]
*Palmaria palmate* ^a, d^	29–46 ^2^			
*Porphyra* sp. ^b, d^	48.6 ± 5.90	18		[7,10]
	40.98			
	35–49 ^1^			
*P. tenera* ^d^	34.7	17.9	16.8	[9]
	12–35 ^2^			
*P. umbilicalis* ^a, d^	43.0 ^c^	34	9	[11]
	29–35 ^2^			
*P. yezoensis*	30–59 ^2^			
*Porphyridium* sp.	35.5 (biomass)	8.5	27	[14]
	45 (EPS)	37	8	
*Pterocladia capillacea*	52.08			[7]
*Sphaerococcus coronopifolius*	41.25			[7]
**Green seaweeds**				
*Caulerpa lentillifera*	38–59 ^2^			
*C. racemosa*	33–41 ^2^			
*Codium reticulata*	39–67 ^2^			
*Enteromorpha* spp. ^a^	33.4	17.2	16.2	[7]
*Ulva* sp.	38^1^	21	17	
*U. compressa* ^a^	41.16			[7]
	29–45 ^2^			
*U. lactuca* ^a^	55.4 ± 2.00			[9,11,13,15]
	38.1–43 ^c^	21.3–24	16.8–19	
	29–55 ^2^			
*U. pertusa* ^a^	52.1			[16]
*U. reticulata*	65.7 ^2^			
*U. rigida*	38–41 ^2^			
**Foods**				
Apple	2 g/100 g	0.9 ^3^		[9,17]
	14.2% DW			
Bean	3.0 g/100 g ^3, e^	0.2		
Brown rice	3.8% DW			[18]
Cabbage	2.3–2.9 g/100 g ^e^	0.3 ^3^		[9,17]
	34.3% DW			
Lentils	8.9 g/100 g			[17]
Rye	20.5 g/100 g ^3^	4.8		
Wheat bran	85% DW			[9]

^a^ approved as edible in France [9]; ^b^ considered as edible by Dawczynski *et al.* [10]; ^c^ values were recalculated in order to be presented in DW basis; ^d^ indicated as edible by McLachlan *et al.* [19]; ^e^ cooked; ^1^ data compiled by Kraan [6]; ^2^ data from several research groups were compiled by Pereira [20]; ^3^ data collected by Praznik *et al.* [21].

### 1.3. Dietary Fibers—Health Benefits

A high portion of dietary fibers regulates transit time, but delays stomach emptying, thus improving nutrient and mineral absorption and retarding hunger pangs. It reduces blood cholesterol as well [21]. In addition, high amounts of fibers in the diet improve the levels of blood glucose, also regulating insulin secretion. This behavior prevents the appearance of postprandial glucose peaks, which would trigger an increase in circulating insulin in order to decrease glucose concentration in the blood. Therefore, intake of dietary fibers would be of additional benefit for patients with diabetes type II [22]. Besides regulating blood glycemic and insulin levels, the soluble fibers also interfere with enterohepatic circulation: by binding to free bile acids or salts, the uptake of dietary fibers promotes their release together with feces. Then cholesterol is used to restore bile, which makes total cholesterol levels decrease up to 18%, this decrease being more obvious for low density lipoprotein (LDL) than for high density lipoprotein (HDL) cholesterol [21,23,24,25]. Additionally, this capacity to absorb bile salts (absorptive capacity) depends on the type and composition of fibers: arabinoxylans have one of the highest absorptive properties, followed by β-glucans and alginates. Cellulose and hemicelluloses present the lowest binding capacity. Furthermore, insoluble fibers are important for the peristalsis of the colon. They can increase bulk weight, capture/hold water and increase the viscosity of stool [21].

### 1.4. Techniques to Obtain Oligosaccharides

Oligosaccharides are LMW-PS that may present a degree of polymerization (DP) of 2–9, sometimes 8–20, monomers. Some researchers consider their DP up to 25 sugar residues [26]. These oligosaccharides with a low DP and a specific sequence of monosaccharides seem easier for fermenting by gut microbiota.

Some techniques have been developed in order to hydrolyze long-chain, branched, diverse PS, which can present different anomeric (α or β) and isomeric (*cis/trans* or *Z/E*) structures. Different enantiomeric forms (*S* or *R*) may also appear, as well as configurations (d- or l-). The types of glycosidic bonds were taken into consideration, as some of these links can be broken by enzymatic action, while others cannot. Some others could eventually be enzymatically severed, but no natural enzymes are known to do it [27,28,29]. Therefore, other techniques have to be chosen so that PS can be hydrolyzed to oligosaccharides. These were reviewed and explained by Courtois [26], who considered several PS and the advantages and disadvantages of each technique. For example, ultrasound was already used for xylan, carrageenan and agarose; microwave was used for the EPS from *Porphyridium cruentum*; free radical depolymerization was used for a fucoidan and a galactan from seaweeds; and other procedures include hydrolysis by concentrated or diluted acids, such as phosphoric acid, and enzymatic hydrolysis [30,31,32,33,34,35,36].

Most acids used do not find application, except for PS constituted by neutral sugars, including fucoidans, carrageenans or galactans. Furthermore, some of the components may be lost during the procedure, and most of the glycosidic bonds are not specifically broken by this method, giving rise to various LMW derivatives [26]. Nevertheless, by using the phosphoric acid to hydrolyze the PS from *Chlorella vulgaris* and *Spirulina platensis*, Leal [34] observed good results for the oligosaccharides as prebiotics. These oligomers enhanced the growth *in vitro* of beneficial bacteria *Bifidobacterium animalis* and *Lactobacillus casei*. Therefore, hydrolysis by the phosphoric acid could be good to use when uronic acids are the main constituents of the PS, as other acids, such as HCl or trifluoroacetic acid (TFA), cannot be used to degrade high molecular weight (HMW)-PS into lower-molecular-weight fractions [26]. On the other hand, agar or agarose-derived oligosaccharides (AGAROS), for example, have been obtained by thermal-acidic hydrolysis with diluted HCl of α-l-anhydrous galactose bonds to 2–6 DP [37].

Free radical depolymerization can also be used to degrade HMW-PS into oligomers, by controlling both the temperature and duration of the process, in order to avoid unwanted ring degradation products [38]. This is usually performed with Cu^2+^ (or Fe^2+^) and H_2_O_2_. Nardella *et al.* [35] found that LMW fucoidans (7.8 and 8.3 kDa) can be obtained by this technique, but other procedures had to be applied in order to improve the former methodology and further fractionate and purify the degraded products obtained. These researchers verified that LMW fractions obtained by anion-exchange chromatography presented lower activated partial thromboplastin times (APTT), meaning that the anticoagulant properties were improved when compared to the native PS [35]. Additionally, this technique does not seem to affect the backbone structure [26].

Other types of techniques include microwave. LMW-PS (9.3 and 15 kDa) were obtained from natural λ-carrageenans of *Chondrus ocellatus* by this technique. These LMW-carrageenan derivatives presented the highest immunomodulatory properties and the greatest antitumor activity in comparison to the native polymer [39]. Eventually, not only the lower MW but also a higher solubility of the obtained fractions contributed to the enhancement of those beneficial properties due to the decrease of the viscosity. Zhou *et al.* [39] also demonstrated that the total sugar and sulphate content of the oligomers did not change upon the microwave treatment. The chemical structure did not present any significant changes as well. Furthermore, when Sun and co-workers [40] submitted the PS from *Porphyridium cruentum* to the microwave technique, they found that the lower-molecular-weight oligomers presented a higher sulphate content. This feature seemed to positively influence the immunomodulatory properties of the LMW-PS derivatives. Some chemical procedures might be used to degrade HMW-PS, the solvents used, or their residues, and may be toxic to the body if the oligosaccharides are to be used in medicine or therapeutics. The microwave technique has at least two advantages: it is not toxic and it is effective from the points of view of energy and time consumption. Ultrasound, another physical technique that can be used to avoid solvents, has the potential advantage of maintaining the initial main chemical structure while obtaining LMW-PS derivatives with controlled molecular weights and conformations [30].

In summary, it seems that physical techniques may be of best use as they may present lower (or no) side effects, as was mentioned by Sun *et al.* [40] and other research groups. In addition, different oligosaccharides with only slightly different structures, obtained by different techniques, have significantly different biological effects [41]. The alteration of the prebiotic potential by using a specific technique may improve the potency of natural prebiotics.

Some other PS-derived oligosaccharides, such as galacto-oligosaccharides (GOS) and xylo-oligosaccharides (XOS), can eventually be obtained from algae, as their PS (xylans, galactans) may contain similar monosaccharide sequences.

We can foresee that it will be possible in the future to modulate the microbiome through drugs and prebiotics. In this way, it will be possible to prevent certain diseases. Wang *et al.* [42] showed, for the first time, that a dietary supplementation with a compound naturally abundant in olive oil and in red wine was able to modulate the microbiome. It prevented gut microbes of mice from turning choline (present in meats, eggs and dairy products) into metabolic by-products, which may lead to atherosclerotic lesion [42]. FOS and inulin (50:50) also influenced the free fatty acid profile of cheese, especially the conjugated linoleic acid. The increase of the conjugated linoleic acid content during the ripening time suggests that a better quality product with lower atherogenicity index may be obtained by the addition of prebiotics during probiotic cheese manufacture [43].

### 1.5. Health Benefits of Algal PS

Concerning the health benefits observed in various animal models and also in humans provided by both algal biomass and PS and LMW-PS from algae, including anti-inflammatory and immunomodulatory properties and several other biologic activities, extensive reviews were recently published [44,45,46]. For example, the biomass and/or derived products from *Arthrospira* and *Nannochloropsis* have been shown to present hypocholesterolemic properties, and *Nostoc* biomass improves digestion and has immunomodulatory characteristics among other biologic activities [44,47,48]. These properties were tested by carrying out both preclinical and clinical trials, using either the algal biomass or some compounds they produce. Related studies were recently reviewed [44] (Table 2).

**Table 2:** Health benefits and basic structures of some algal PS (native or somehow modified, such as LMW-PS) similar to dietary fibers [45,46].

Polysaccharide/LMW-PS	Health Benefit	Main Glycosidic Linkages and Monomers along the Main Chain	Algal Genera
sPS	Antilipidaemic/hypocholesterolaemic		*Porphyridium* (R), *Rhodella* (R)
s-laminaran		(1,3)- and (1,6)-β-glc	*Ascophyllum* (B), *Fucus* (B), *Laminaria/Saccharina* (B), *Undaria* (B)
s-fucan			*Sargassum* (B)
s-galactofucan		(1,3)- and (1,4)-α-l-fuc (alternating)	*Laminaria/Saccharina* (B),
s-galactan (porphyran)		(1,3)-β-d-gal or (1,4)-α-l-gal	*Porphyra* (R), *Ulva* (G)
s-ulvan		(→4)-β-d-GlcAc-(1,4)-α-l-rham3S-(1→)	*Ulva* (G), *Enteromorpha* (G)
		(→4)-α-l-IduAc-(1,4)-α-l-rham3S-(1→)	
sPS	Antiglycaemic		*Porphyridium* (R), *Rhodella* (R)
(s)PS	Immunomodulatory		*Chlorella* (G), *Gracilaria* (R), *Gyrodinium* (Dino), *Phaeodactlylum* (Diat), *Porphyridium* (R*), Ulva* (G)
s-fucan		(1,3)-α-l-fuc	*Cladosiphon* (aka *Okinawa)* (B)
s-fucan		(1,3)- and (1,4)-α-l-fuc (alternating)	*Ascophyllum* (B), *Fucus* (B)
s-laminaran		(1,3)- and (1,6)-β-glc	*Ascophyllum* (B), *Fucus* (B), *Laminaria* (B), *Undaria* (B)
s-galactofucan		(1,3)- and (1,4)-α-l-fuc (alternating)	*Laminaria* (B), *Undaria* (B)
s-ulvan		(→4)-β-d-GlcAc-(1,4)-α-l-rham3S-(1→)	*Ulva* (G), *Enteromorpha* (G)
		(→4)-α-l-IduAc-(1,4)-α-l-rham3S-(1→)	
(s-) rhamnan			*Enteromorpha* (G), *Monostroma* (G)
LMW-sPS			*Furcellaria* (R), *Soliera* (R)
LMW-carrageenan		(1,3)-α-d-gal, and (1,4)-β-3,6-Agal or (1,4)-β-d-gal (alternating)	*Kappaphycus* (R)
s-mannan			*Capsosiphon* (G)

B, brown; CB, cyanobacteria; G, green; Diat, diatom; Dino, dinoflagellate; R, red; Agal, anhydrous galactose; fuc, fucose; gal, galactose; glc, glucose; glcAc, glucuronic acid; IduAc, iduronic acid; rham, rhamnose; sPS/-other, sulphated PS (in general) or any specific PS .

### 1.6. Algal PS as Dietary Fibers

Despite the high carbohydrate contents in marine algae with 25%–75% of the dry weight of seaweeds, most of them are not digested in the human gastrointestinal (GI) tract, as it was inferred before [6,49,50,51,52]. Therefore, they act as dietary fibers, some being soluble fibers (50%–85%: agars, alginates, fucoidans, furonan, laminaran, porphyrin, ulvan, carrageenans, xylan) that are not fermented, at least not completely, by colonic microbiota to short-chain fatty acids (SCFAs) [6,9,49]. Additionally, soluble fibers have the capacity to pass along the GI tract without being metabolized, and, due to their viscosity, they slow down digestion [18]. However, they decrease nutrient absorption, since minerals and other nutrients may adhere to the fibers by chelating with them, thus decreasing their availability. Beta-glucans, such as those from *Chorella* and *Undaria*, were also suggested to be able to regulate postprandial levels of glucose and insulin [53]. Some other algal PS can act as insoluble fibers (cellulose, mannans, part of xylans, part of alginates, β-glucans, and lignin). They may interfere with mineral and protein absorption while decreasing transit time [6,21]. These insoluble fibers increase fecal stool bulk due to their capacity to hold water [24,25].

The highest insoluble fiber content is found in *Fucus* (40%) and *Laminaria* (27%), two brown seaweeds, while *Undaria* (B), *Chondrus* (R) and *Porphyra* (R) are the richest genera in soluble fibers: 30%–33%, 15%–22%, 18%–34%, respectively [54] (Table 1). *Palmaria palmata* may include up to 35% DW in β-d-xylans, a soluble fiber [55].

Alginates, for example, are important as soluble dietary fibers, as HMW-alginates (≥50 kDa) prevent obesity by decreasing body weight, as was demonstrated in clinical trials. Alginates are also able to reduce cholesterol and to prevent diabetes, especially by avoiding postprandial peaks of glucose and insulin. The levels of *C*-peptide were lowered as well, and gastric transit was delayed. Alginates and related oligosaccharides have demonstrated *in vivo* prebiotic properties as well, by promoting fecal microbiota metabolism in humans [56,57,58,59,60,61].

Carrageenans, also soluble fibers, can regulate the metabolism of the gut by treating and preventing dysentery, constipation and diarrhea. They can prevent diabetes and associated diseases as well [62,63].

Laminarans (or laminarins), found especially in *Laminaria/Saccharina*, were recognized as dietary fiber with prebiotic properties, with applications as substrate for probiotic bacteria [64]. Furthermore, laminaran seems to play a role in regulating the gut metabolism by interfering with gut goblet cells of the epithelial layer and stimulating the release of mucus. It decreases the intestinal pH and enhances the production of SCFAs [6,65]. Laminaran can also act as immunomodulator by stimulating B- and helper T-cells, and by regulating the cholesterol levels both in the liver and in the blood, as these polymers are able to inhibit the absorption of cholesterol in the gut, this compound being released in the feces [6]. Laminaran can reduce glucose levels as well. In *Palmaria palmata*, the soluble prebiotic fibers are fermented by colonic bacteria into SCFAs, as reported two decades ago by Lahaye *et al.* [66].

In a study conducted by Dvir and colleagues [14], the PS from *Porphyridium* was fed to Sprague-Dawley rats, causing a significant increase in the fecal stool bulk of the animals, while GI transit time decreased. This reduction was even more relevant in the animals fed algal biomass (51%–60% reduction). Soluble EPS from *Porphyridium* were also able to reduce blood lipid levels and cholecystokinin, both in the plasma and in duodenal mucosa, and shifted up fecal excretion of bile acids and neutral sterols. Furthermore, besides causing metabolic changes, PS from this red marine microalgae could induce morphological modifications in the small intestine and colon of the treated animals. A notable increase in the number of goblet cells in the mucosa layer was observed with the consequent enhancement of viscosity of fecal contents. Morphology of the *tunica muscularis* of the jejunum was enlarged. It seems that those morphological modifications may happen so that nutrient and mineral malabsorption might be overcome [14]. However, the effects on the SCFA production and on the gut microbiota were not determined. Therefore, some more studies are necessary in order to evaluate the prebiotic properties for animals and humans.

Additionally, algal fibers already proved not to be toxic [29,67]. Furthermore, some of the PS produced by marine algae (alginates, agars, carrageenans, fucoidan, mannitol, laminaran, ulvan) and/or their biomass can be considered as functional foods, as they confer specific health benefits other than the “simple” nutrition [6,45,68]. These benefits include antiviral capacity, prevention of cancer, obesity and diabetes, decrease of total and LDL cholesterol, and also postprandial glucose levels, which are some of the chronic diseases associated with a low consumption of dietary fibers [6,45,46,69,70].

## 2. Prebiotics

### 2.1. Definitions and Criteria

Sometimes the concepts of “dietary fiber” and “prebiotic” are understood as being synonyms. In 1993, Roberfroid [71] proposed, at first, a definition of prebiotic, which two years later was widened to a “non-digestible food ingredient that beneficially affects the host by selectively stimulating the growth and/or activity of one or a limited number of bacteria already resident in the colon, thus improving the host’s health” [71,72]. A decade afterward, this group of researchers redefined the concept as “selectively fermented ingredient that allows specific changes, both in the composition and/or activity in the GI microflora that confers benefits upon host well-being and health” [73]. This implies that changes of the microbiota may occur along the entire GI tract and not only in the colon. Thus, this definition was further improved so that a prebiotic is actually a “selectively fermented ingredient that results in specific changes in the composition and/or activity of the GI microbiota, thus conferring benefits upon host health” [74]. Additionally, Gibson *et al.* [73] established the criteria for a food ingredient to be accepted as a prebiotic: (i) the substance must be somewhat resistant to the acidic and enzymatic digestion, and also to the absorption in the upper part of the GI tract; (ii) it should be fermentable by the large intestine microorganisms (microbiota); (iii) it must selectively stimulate the growth of those (indigenous) bacteria in the gut and/or the effects provided by their activity, which enhances health and well-being of the host. However, to fulfill this criterion, the interactions between the various species and groups of bacteria must be safeguarded, as colon microbiota is a very complex “ecosystem”, comprising both aerobic and anaerobic, bifidobacteria and lactobacilli, among others [75]. In addition, this has to be confirmed *in vivo* by carrying out proper clinical trials, and not only *in vitro* tests, even when bioreactors are used to simulate bacterial activity in the colon.

According to Binns [76], regardless of the benefits observed in animal models and results obtained *in vitro*, some clinical trials with humans must be performed during a certain period of time and within acceptable doses, and the health benefits for the hosts must be demonstrated in order for a candidate to be considered a prebiotic. With respect to seaweeds and microalgae, perhaps this is the “only step required” for most PS produced by these marine organisms to be accepted as prebiotics. In addition, it must be highlighted that some oligo- and polysaccharides (GOS, XOS, xyloarabinans, galactans, β-glucans), already accepted as prebiotics, are part of algal PS. Furthermore, although the prebiotic character was not the focus until now, PS from algae have already been subjected to some clinical trials in humans, as was recently reviewed [44].

Gibson *et al.* [74] suggested that prebiotics serve as substrates to be degraded by the enzymes of colonic microorganisms. These prebiotics include resistant starches, dietary fibers (usually PS, DP > 10), oligosaccharides, some non-absorbable sugars and sugar alcohols, proteins and amino acids, and other materials, including mucins, bacterial metabolites and products from cell lysis. It is known that both macro and microalgae are rich sources of most of these compounds, some of them already demonstrated to possess prebiotic properties as well.

### 2.2. Benefits of Prebiotics to Human Health

The microbiota profile associated with the production and use of SCFAs may be affected by age, as well as immunity status and stress condition, which might also have an influence on the colonic transit time [77]. Lower levels of SCFAs in healthy individuals’ feces may be an indication of a better absorption rather than a lower production [78]. In fact, most of the SCFAs (acetate, butyrate, and propionate) that rise in the large intestine as metabolites from bacterial fermentation enter the systemic metabolism through the blood circulation [79].

**Figure 1:**
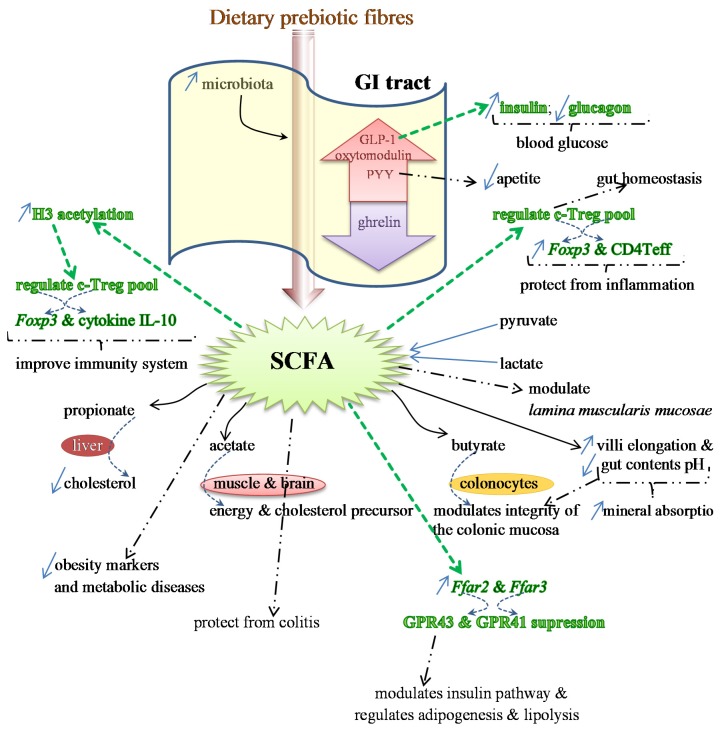
Effects of SCFAs on the expression of several genes, which regulate the production of different proteins and some anti-inflammatory cytokines (colored in green) involved in the immunity status and protection from inflammation. SCFAs also regulate the release of GLP-1, responsible for the increase in the production of insulin and decrease in glucagon (in green), which reduces blood glucose levels. Additional benefits provided by SCFAs are indicated by bold dashed arrows. Specific properties of the main SCFAs (propionate, acetate and butyrate), and the target organs are illustrated as well.

Other products, such as pyruvate and lactate, can also be further transformed into SCFAs [80]. This is the reason for these organic acids not being found in high amounts in the large intestine when searching for the end-products of the metabolism of a prebiotic (fiber). These SCFAs and lactate are known for their beneficial effects. For example, propionate is used in the liver, where it was suggested to exert some function on cholesterol synthesis, thus decreasing its levels in the liver and in the blood. On the other hand, acetate is transformed by muscle and brain cells and may be a precursor for cholesterol synthesis, while butyrate is metabolized in colonic cells. Here it seems to play a role in maintaining the integrity of the colonic mucosa by positively interfering in the activity of epithelial cells and also in cell apoptosis [21,81,82] (Figure 1). Additionally, there could be a significant increase in total SCFAs, but when they are considered individually, such differences may not exist for each of the SCFA levels.

However, the end-products of proteolytic or peptolytic fermentation (amines, phenols, ammonia) may exert harmful effects by triggering some diseases and bowel disorders [83]. Nevertheless, health-promoting bacteria of microbiota may exert positive effects on such diseases (IBDs and irritable bowel syndrome, IBS) [76]. Some toxins may also be produced [74].

Some of the benefits attributed to bifidobacteria and lactobacilli include immunomodulation, improvement of digestion and absorption of nutrients and minerals, reduction of cholesterol and glycemic indexes, reduction of bloating and eructation, and shift down of the levels of putrefactive metabolites [72,76,84,85,86] (Table 3). Other health benefits attributed to probiotics include the improvement of the lactose tolerance (in celiac individuals) and a beneficial shift in microbiota (not always reported, though) that are purportedly able to inhibit the growth of harmful bacteria, and to prevent cancer [74,87]. In addition, probiotic bacteria protect against gastroenteritis. Also, prebiotics will eventually replace the antibiotics used as growth stimulants in apiary, fishery, poultry and animal husbandry [36].

Gibson and colleagues [74] compiled an interesting list of relevant studies on the effects of some known prebiotics. However, all the studies were short-term and low-number tests. Most subjects indicated in Gibson’s review were volunteers and/or healthy individuals; studies with rats were also included. Several techniques were used to analyze the results of each of the studies [74] (Table 3).

**Table 3:** Effects attributed to pro- and prebiotics, and mechanisms through which benefits are exerted.

Effect	Mechanisms	References
IBD ^a, b^	reduction of pro-inflammatory immune markers and also of calprotectin enhancement of cytokine production reduction of symptoms modulated by bifidobacteria (highest benefits usually when butyrate is used in the experiments)	[21,36,74,82,88,89,90,91,92,93]
Ulcerative colitis ^a, b^		
Pouchitis ^b^		
Crohn’s disease ^b^		
IBS ^a^		
Colon cancer, prevention	significant reduction of putrefactive compounds	[74,88,93,94,95]
	production of butyrate to act as protective agent	
	mediation by colonic microbiota, as bifidobacteria may shift down carcinogenic promoters and genotoxins	
	reduction on biomarkers for cancer	
	reduction of cell proliferation	
Bone mass/density	enhancement of calcium absorption ^a, b^, due to the release of SCFAs	[21,74,88,96,97,98]
	shift down of gut pH due to the production of SCFAs	
Regulation of gut metabolism/transit	reduction of constipation of diarrhea and dysentery	[74]
Antibiotic-associated and traveller	reduction of the prevalence of/prevention from diarrhoea ^b^ reduction of the fever and vomiting in children ^b^ stimulation of the growth bifidobacteria	[93,99,100,101]
diarrhoea		
Improvement of the immune system	production of pro-inflammatory cytokines (TNF-α)	[74,102]
	expression of receptors on macrophages and lymphocytes T and B are stimulated	

Other effects of prebiotics are promising in lipid metabolism, as hypoglycemic, immuno-modulators, regulating release of hormones in the gut, weight loss and increase of satiety sensation. Gibson *et al.* [74] published a review on this subject. ^a^ studies with animals; ^b^ studies/trials with humans; IBD = inflammatory bowel diseases; IBS = irritable bowel syndrome; TNF = tumour necrosis factor.

#### Benefits of Prebiotics Reflected in the Morphology, Ecology and Microbiota of the Gut

As dietary fibers enhance fermentation in the colon and improve gut microbiota, they may provide protection against obesity and associated metabolic diseases [77]. The main end-products of dietary fiber fermentation by colonic bacteria are SCFAs, such as acetate, propionate and butyrate, whose concentration and pattern along with bacterial diversity and number may be influenced by the type and amount of ingested fibers [103].

As it happens with diet differentiation, which modifies the microbiota profile, lean (LN) and normal subjects’ microbiota differs from overweight and obese (OWOB) individuals’, either in animal models or humans. Additionally, this may be one of the causes of obesity: a higher production of SCFAs is concomitant with an energy release that can be absorbed and used again by OWOB microbiota [77,104,105]. However and despite the increase in fecal SCFA levels, the Firmicutes:Bacteroidetes (F:B) ratio in OWOB people also increases with body mass index (BMI) [77]. This could be confirmed as a decrease in the F:B ratio was associated with a decrease in obese subjects’ body weight [106]. Nevertheless, the microbiota profile may differ between individuals, which may interfere with the bacterial numbers of same species or genera, creating a false contradiction between the F:B ratio and LN:OWOB people ratio. Furthermore, the size of the tested population and the analytical methods used may also contribute to this discrepancy, and so does the region of sampling (in feces or still in the distal portion of the colon or rectum). This last parameter has to be considered, as higher levels of SCFAs in feces may not necessarily mean an increased production by gut microorganisms. They may be due to a decrease of absorption by OWOB individuals, as obese patients usually have some other metabolic disorders and diseases, which may be associated with a lower number of microorganisms that use the excess of SCFAs produced.

As Ley *et al.* [104] showed, obesity causes modifications in the gut ecology of microbiota either in humans or mice. In addition, both diversity and number of gut microorganisms that colonize the GI tract change during severe periods of diarrhea, including those provoked by cholera, antibiotics or rapid weaning of changes to normal diets [107,108,109,110]. However, supplementation with prebiotics can prevent the proliferation of pathogenic bacteria as the increase in SCFAs and other organic acids may lower pH, destroying *E. coli* and/or *Salmonella* [111,112]. Further, the administration of SCFAs during dysentery stabilizes fluid losses and restores the levels of beneficial-to-harmful bacteria in the GI tract. The consumption of prebiotic CHO enhances the numbers of bifidobacteria and other indigenous beneficial organisms while maintaining total bacterial counts [76,113,114,115,116,117].

In summary, it seems that prebiotic administration can exert its effect either through the production of SCFAs, and the consequent decrease in pH, or by promoting the adherence of the bacteria to those CHO, resulting in the elimination of the pathogenic microorganisms and/or preventing them from translocating across the epithelial cells of the GI tract [76,108]. Colon ecology and morphology may also be changed by other dietary components, such as fatty acids, which may modify gut microbiota by improving or inhibiting microbial adhesion (and growth) to the intestinal wall [76] (Figure 1).

With respect to the morphology of the GI tract, several studies with different animal models resulted in positive results on the villi length and intestinal pH. Prebiotics, either oligosaccharides or native PS, caused an increase in the mucosa layer of the animals, with an elongation of the microvilli, and an increase in the number of epithelial cells. They caused a decrease in the intestinal pH as well [14,118,119,120,121,122,123,124]. These changes of the gut morphology and chemistry created an enhancement of the surface area and the acidic conditions adequate to promote mineral absorption (Figure 1). This improvement is due to the formation of mineral soluble complexes, but it may also be associated with an increase in the permeability of the cell membrane of the enterocytes [120]. Additionally, the ingestion of prebiotic and/or symbiotic diets also caused significant differences in anastomosed animals. In fact, they presented a higher number of goblet cells and elongation in both villi and crypts, and also an enlargement of the *lamina muscularis mucosae* [119,125,126]. These morphological changes provided the animals with functional characteristics that were removed during the surgery [119].

As a whole, the improvement of the function of the epithelial cells surrounding the internal wall of the intestines and the histomorphology of the GI tract, together with the immunology of the mucosal layer, seem to be important for providing benefits and maintaining a general healthy status [127]. Some researchers state that this ability, along with the probiotic properties of some beneficial bacteria, might be due to an improvement of the cell cytoskeleton and of the protein phosphorylation at the tight junction level [128].

### 2.3. SCFAs—Molecular Mechanisms and Epigenetic Regulation

SCFAs are end-products of the metabolism of prebiotics that have proven to restore the number and frequency of the colonic Treg pool, as well as protecting from colitis. This protection is dependent on the expression of *Ffar2*, the genes that encode for G-protein receptor 43 (GPR43) or free fatty acid receptor (FFAR2). The expression of this protein receptor, together with GPR41 (or FFAR3), is modulated by SCFAs. The activation by acetate and propionate is stronger than with butyrate [129,130]. GPR43 is expressed in various tissues and is involved in the regulation of inflammatory processes, immunity and homeostasis [93,131] (Figure 1).

By suppressing the GPR43 expression in the ileum and colon of both rats and humans, SCFAs interfere in the adipogenesis, lipolysis and insulin pathway, and also in the secretion of the gut hormones peptide YY (PYY) and proglucagon protein-1 (GLP-1) [131]. It is considered that dietary fibers may reduce the risk of obesity, and this may be associated with the increase of SCFA production. This stimulates the secretion of gut hormones elicited by specific cells of the mucosa layer, such as PYY, GLP-1 and oxytomodulin. SCFAs also inhibit the release of ghrelin and provide protection against obesity [76,93,131,132,133,134,135,136]. PYY is usually involved in the reduction of appetite; GLP-1 stimulates the insulin production and inhibits the glucagon release, hence lowering the levels of blood glucose. Therefore, either GPR43 or PYY and GLP-1 may be potential drug targets for therapeutics of obesity and type 2 diabetes and associated diseases in order to control the appetite and/or the glucose tolerance [131] (Figure 1). Detailed information on the roles of SCFAs in the genes’ expression of GPR43 and GPR41 and in the hormones PYY and GLP-1 may be obtained in two reviews by Kimura *et al.* [131] and Kasubuchi *et al.* [93].

Colonic Treg (T regulatory) cells, whose responses are stimulated by the activity of *Bacteroides fragilis* and Clostridium, are responsible for the maintenance of the homeostasis in the intestine and protect from inflammation by expressing the transcript factor Foxp3 and augmenting the effector CD4 T cells (Teff) [137,138]. SCFAs also interfere positively on the health of the immune system, as it was confirmed in germ-free mice models. These animals usually present deficient immune responses, including the c-Treg pool, due to low levels of acetic, propionic and butyric acids in their intestine [139,140]. This deficiency may be mitigated by feeding animals SCFAs, which regulate the number of Treg cells through the stimulation of histone H3 acetylation [93]. This regulation enhances the expression of the anti-inflammatory cytokine IL-10 (interleukin 10) and Foxp3 [139,140] (Figure 1).

SCFAs can also enhance the expression of G-protein receptors, at least in mice and rats [131]. This and other health beneficial effects caused by fermentation of SCFAs in the gut were studied and reviewed by other researchers as well, including Smith and colleagues [140]. SCFAs seem to exert their effects either directly within the intestinal *lamina propria*, after diffusion across the epithelium, or indirectly by entering into the epithelial cells of the colon internal wall [140,141,142].

SCFAs may also exert their role by inhibiting the enzyme histone deacetylase (HDAC) [140]. These short-chain carboxylic acids, which are produced in the colon by microbial anaerobic fermentation, are promising as chemoprotective agents, in special butyrate, and propionate and valerate as well. They exert their anticancer activity by stimulating protein-p21 and (p21)-gene expression, and by suppressing protein cyclin CB1 through the inhibition of HDAC. Henceforth, they promote the hyperacetylation of histone-associated proteins H3 and H4 [143,144,145]. It is worth noting that the cell-cycle regulator protein p21 is an inhibitor of kinases (CDK) that are cyclin-dependent [146]. In addition, protein p53, which is known as a tumor-suppressor, can inhibit the mitosis-regulator protein CB1. However, it seems that inhibitors of HDAC, such as butyrate, must bind to some specific proteins, Sin3 and NuRD, in order to be able to deactivate the enzyme [143].

Nevertheless, the gene expression, such as the one for intestinal alkaline phosphatase (IAP, a differentiation marker), may also be modulated following other mechanisms. Butyrate seems to be associated with the modulation of phosphorylation of some proteins, and methylation of both proteins and DNA [93,147]. In addition to the high ability to inhibit HDAC, butyrate can induce differentiation and apoptosis of either precancerous or neoplastic cells, besides being a strong antiproliferative agent as well. Furthermore, butyrate can promote hyperacetylation, which leads to apoptosis, together with the activation of the proenzyme caspase-3 into caspase-3 protease, which is its active form [143,144,148,149]. Additionally, the hyperacetylation of histones interferes with genes’ transactivation by regulating transcriptional factors, and with the expression of other genes, such as IAP [150].

Various other studies on the effects of SCFAs, as HDAC inhibitors, on the differentiation and apoptosis of several cancer cell lines were reviewed by Meijer *et al.* [151]. According to these researchers, besides this inhibition, SCFAs in general and butyrate in particular seem to exert an anti-inflammatory effect through another signaling pathway, associated with the nuclear factor-κB (NF-κB). By preventing the translocation of NF-κB into the nucleus, SCFAs are also able to suppress the expression of some pro-inflammatory genes, such as cytokines TNF-α, IL-1β and IL-6 [151]. Additionally, butyrate can also inhibit the expression of the pro-inflammatory cytokine IFN-γ, thus leading to the improvement of the inflammation process as a consequence of the reduction of inflammatory markers. In contrast, propionate and acetate showed an increase in IFN-γ release [152]. However, curiously, when a mixture of these three SCFAs is administered, they provide a synergistic effect with a higher release of IFN-γ. Nevertheless, both propionate and acetate can also have a role as endogenous anti-inflammatory agents, as it was reported by several groups of researchers, whose studies were compiled by Meijer *et al.* [151]. The expression of a number of other cytokines is also regulated by short-chain carboxylic acids, especially butyrate, their release and effects being dependent on the dose administered [151].

## 3. Seaweeds and Marine Microalgae and Their Active Compounds as Prebiotics

Some (candidate) prebiotics occur naturally in seaweeds and marine microalgae, and some of their PS (native or somehow modified, such as LMW-PS) were already recognized and accepted as dietary prebiotics: GOS, AGAROS, XOS, neoagaro-oligosaccharides (NAOS), alginate-derived oligosaccharides (ALGOS), arabinoxylans, galactans, β-glucans, although the fulfillment of the criteria still has to be proved for some of them. However, these algal PS are not degraded by enzymes in the upper part of the GI tract. Therefore, they can be used as dietary prebiotics (fibers), as they also enhance the growth of lactic acid bacteria (LAB) [29].

### 3.1. Brief Description of the PS Considered as Fibers

An extensive review on the PS from marine algae was recently published [45]. The authors focused on the overall structures of the PS from the various big groups of seaweeds (phaeophytes, rhodophytes and chlorophytes) and the most-studied genera of marine microalgae, including some cyanobacteria. Additionally, the monosaccharide compositions and the linkage types were described, as well as some of the di- and oligosaccharides that were already referred to as being part of the PS of some microalgae. For example, brown seaweeds contain mostly fucoidans, soluble homo- or heteropolymers, with l-fucose as the main sugar residue; fucoidans are irregularly branched sulphated HMW-PS, whose monomers are usually linked by (1,3)- and (1,4)-α (alternating) bonds.

Alginates are the principal carbohydrates in *Ascophyllum*, *Fucus* and *Sargassum* (20%–29% DW), which may also present fucoidans in lower percentages (10%–11% DW) [153,154,155]. *Laminaria/Saccahrina*, *Ascophyllum*, *Fucus* and *Undaria* also contain laminaran, a β-glucan, with (1,3)- and (1,6)-β-glucose linkages, with some other sugar residues linked laterally. Galactofucans may appear in some brown macroalgae (*Laminaria*, *Undaria*) as well [45].

Alginates are anionic-acidic, non-branched soluble PS, already used in the food industry (E400–E407), whose monomers are l-guluronic acid (or guluronates) and d-mannuronic acid (or mannuronates) [156]. Alginates appear mainly in *Laminaria* and *Macrocystis.*

The main carbohydrates of red seaweeds are floridean starch (as reserve/storage) and S-galactans (carrageenans and agarans), as is the case of *Chondrus* and *Kappaphycus*, and *Porphyra* and *Gracilaria*, respectively. Usual linkages and principal monomers are (1,3)-α-d-galactose, and (1,4)-β-3,6-anhydrous galactose or (1,4)-β-d-galactose (alternating). As it was mentioned earlier, AGAROS can be obtained by acid-hydrolyzing α-l-anhydrous galactose bonds [37]. These oligosaccharides were already showed to provide several health benefits [21]. Some genera may present xylomannans (*Sebdenia* and *Nothogenia*), and xylogalactans (*Nothogenia*) as well.

Carrageenans are widely used in foods, for example, as gelling agents in plant-derived gelatines. Polysaccharides from green seaweeds may also consist of (gluco)mannans (*Capsosiphon*) and a rare (1,3)-β-mannan in *Codium* *fragile*, while ulvan is the main PS present in green macroalgae (*Enteromorpha*, *Ulva*). Rhamnans (*Enteromorpha*), galactans (*Caulerpa*) and other, more complex PS may appear as well [45].

In what concerns PS from microalgae, there is not much information on these complex polymers. Except for a β-glucan in *C. vulgaris* and a homogalactan in *Gyrodinium*, most of the other PS are heteropolymers of several different monosaccharides. The glycosidic bonds were described for only a few PS, including those from *Aphanothece halophytica* and *Phaeodactylum tricornutum.* However, the structures for the repeating mono-, di- and oligosaccharides were already described for the PS of *Arthrospira platensis*, *Porphyrium* and *Rhodella* [45].

Hemicelluloses may include most of the algal PS, which are usually branched polymers embedded in algal cell walls, present within the cell or even produced and released into the culture medium. These are heteropolymers that can be easily hydrolyzed by hemicellulases or by acid or basic diluted solutions. In addition to the PS that are mostly considered as soluble fibers, seaweeds also contain cellulose, which is a linear non-branched polymer made up by only anhydrous glucose residues linked together by β-(1,4) bonds. Cellulose and lignin are insoluble fibers, resistant to microbial and human enzymes [21].

### 3.2. Prebiotic Benefits of Algal Biomass and Fibers, Oligo- and Polysaccharides

The health benefits and biological activities of polysaccharides produced by both marine macro and microalgae were recently reviewed [45,46]. Additionally, the results of several clinical trials showing the health benefits of marine microalgae biomass intake and microalgal-derived products were also reviewed and indicated in another recent work [44]. Scarce information exists regarding the effects on microbiota, though. Courtois [26] came forward with an explanation for these long-chain HMW-PS to present such prebiotic/beneficial effects at the cell level, most of the time preventing or decreasing reactive oxygen species (ROS) production. This researcher proposed that such HMW-polymers must first be hydrolyzed through digestion or fermented by the gut microorganisms before being assimilated as smaller oligosaccharides and entering the cells, where the oligomers are active [46,157]. After being ingested, algal PS can resist hydrolysis in the upper part of the GI tract, until they reach the large intestine [64,158,159,160].

However, HMW-PS may be hydrolyzed by the enzymes of some colonic microorganisms, *i.e.*, their molecules may be broken apart into mono- and/or oligomers. Some of the best-characterized enzymes produced by such microorganisms—bifidobacteria and lactobacilli—are xylases and (glycosyl) hydrolases (α- and β-galactosidases, α-glucosidase, fucosidase) [29,158,159,161,162]. Some species of bifidobacteria can also hydrolyze arabinans, arabinogalactans, arabinoxylans from plant or algal origin, as, possessing the arabinofuranohydrolases, they are able to ferment arabinofuranosyl-monomers [29]. Those LMW-saccharides are then fermented by other groups of bacteria into SCFAs, which can be monitored by GC-MS, for example [163]. In addition to intestinal bacterial enzymes, laminaran may also be degraded by laminarases and laminarinases [64]. Moreover, before being used, fucoidans may be hydrolyzed into oligomers by fucoidanases, which are enzymes produced by some bacteria and mollusks from marine environments, but not by colonic microbiota [164,165]. Furthermore, there are proofs of the microbiota changes when subjected *in vitro* to the effects of LMW-PS prebiotics from algal origin, such as those from *Gelidium*, and laminaran and alginate/ALGOS, with the formation of SCFAs [61,165,166,167,168]. Nevertheless, and despite the results obtained from *in vitro* studies, some positive effects on the number of lactobacteria were observed in pigs fed fucoidans [166,169,170,171] (Table 4).

**Table 4:** Prebiotic effect of algal biomass, their extracts and oligo- and polysaccharides.

Oligo-/PS	Algal Genus	Effects	*In Vitro/in Vivo* (Animal Model)	References
alginate	-	↑ *Bifidobacterium*	rats	[61]
		*↑ Lactobacillus*		
NAOS (native and hydrolysates, DP 4–12)	-	↑ *Bifidobacterium*	mice/rats (*in vitro*)	[158]
		↑ *Lactobacillus*		
		*↓ Bacteroides* and enterococci		
		*↓* pH in medium		
		*↓* putrefactive microorganisms		
laminaran	-	↑ *Bifidobacterium*	rats (*in vitro*)	[165]
		*↓* putrefactive compounds		
laminaran + fucoidan	-	↑ lactobacilli	weanling pigs	[172]
		*↓* diarrhoea		
extracts	*Undaria/Porphyra*	*↓* enzymes responsible for the transformation of pro- into carcinogens	rats	[173]
biomass	*Ascophyllum*	↑ *Lactobacillus*/*Escherichia coli*	weanling pigs	[174]
biomass/extracts	*Laminaria*	↑ SCFAs	weanling pigs	[175]
		*↓* ammonia in the colon		
fucoidan	-	↑ lactobacteria	pigs	[166,171,176]
		↑ fatty acids		
alginate	-	↑ beneficial bacteria of microbiota	humans	[60]
ALGOS and native or LMW-PSs	*Gelidium*	● positive effects on the microbiota and on the production of SCFAs *↓* putrefactive compounds	rats	[61,165,167,168]
		*↓* putrefactive microorganisms		
FUCOS	-	↑ beneficial bacteria	-	[158,168]
AGAROS	-	*↓* pro-inflammatory cytokines	-	[177,178]
		● act against glycosidase		
extracts	*Gelidium*	↑ bifidobacteria; best with *Gelidium*-extract	*in vitro*	[179]
	*Gracilaria*			
	*Ascophyllum*			
		↑ total SCFAs, and acetic and propionic acids; best with *Gelidium*-extract		
biomass	*Chondrus*	↑ beneficial bacteria	rats	[127]
		● improvement of microbiota		
		↑ SCFAs		
		● improvement in the histo-morphology of the colon		
		↑ holding-water capacity of stool		
		● enhancement of immune system: ↑ Ig-A and G		
biomass	*Spirulina*	↑ *L. casei*, *L. acidophilus*, *S. thermophillus* and other beneficial bacteria, such as *Bifidobacterium*	*in vitro*	[87,180,181]
		*↓* harmful bacteria: *P. vulgaris*, *B. subtilis*, *B. pumulis*		
biomass	*Isochrysis*	↑ lactic acid bacteria	rats	[182]

↑ increase; *↓* decrease.

Additionally and despite the lack of proper enzymes to digest fucoidans in the GI tract, this type of PS was already detected in human blood and urine after oral administration [183]. Therefore, it is highly probable that humans can use and transform fucoidans [29]. Furthermore, the LMW-fucoidan derivative *S*-galactofucan was already detected in human blood after the intake of *Undaria* dried biomass or extracted and purified *S*-galactofucan [184]. In addition, fucoidan from *Laminaria* spp. or their extracts showed prebiotic effects on pigs, with an increase of SCFAs in the colon, and in the number of bifidobacteria and lactobacteria detected in the cecum and large intestine, respectively [175,176]. Nevertheless, most of the *in vivo* results were obtained with rats, through the determination of putrefactive compounds in their feces [61,158,165].

Fucoidan (FUCOS) and ALGOS have also proven to increase the number of beneficial bacteria *in vitro*, with better effects than those of fructooligosacharides (FOS) or lactose, a group of studied prebiotics. Moreover, ALGOS inhibit the growth of putrefactive microorganisms [61,158,168,185].

As it happens with fucoidans, NAOS and AGAROS, before being ingested, should be first obtained by hydrolysis by β-agarases and α-agarases, respectively; α-agarases hydrolyze α-(1,3) bonds, while β-agarases break β-(1,4) linkages [158,186]. However, these two hydrolases are produced by non-colonic bacteria [158]. NAOS hydrolysates with different DP (DP 4–12) were tested in rats by Hu *et al.* [158]. *In vitro* studies conducted by these researchers showed that these NAOS hydrolysates and native NAOS could be metabolized by bifidobacteria and lactobacilli, but the NAOS-B with DP 8–12 were best fermented. A decrease in the pH of the culture medium may be an indication that those beneficial bacteria had better fermented those oligosaccharides. However, SCFAs were not determined. Additional evidence of the prebiotic properties of these oligosaccharides was shown by a significant increase in the numbers of the various species of *Bifidobacterium* and *Lactobacillus*, but not enterococci, which decreased. A positive change in the numbers of beneficial bacteria was also observed *in vivo*, in the feces and cecal contents of rats and mice, and the growth of bacteroides was inhibited together with other putrefactive microorganisms [158]. Hu *et al.* [158] verified that the prebiotic effectiveness of NAOS, especially NAOS-B (DP 8–12), was higher than that of other known oligosaccharides (FOS and GOS) as well. AGAROS had already been proven to be able to inhibit the release of pro-inflammatory cytokines and to act against the enzyme glycosidase [177,178]. The decrease in the pH in *in vitro* studies with different algal-derived oligosaccharides, following an increase of the number of beneficial bacteria (mostly bifidobacteria and lactobacilli), is probably due to the production of SCFAs. However, these end-products of the bacterial fermentation of the oligosaccharides were not determined, the same happening with several animal models [61,158] (Table 4).

A somewhat different *in vitro* study was carried out by Ramnani *et al.* [179]. They subjected human feces (with respective microbiota) to native and LMW derivatives from alginate and agar, along with extracts from *Gelidium*, *Gracilaria* and *Ascophyllum*, and observed that LMW-PS effectively induced changes in the microbiota. However, the effectiveness was greater with *Gelidium* extract, with a significant increase in the number of bifidobacteria. Furthermore, a shift up in SCFAs was observed with a significant increase in acetic and propionic acids after fermentation of the oligo- and polysaccharides from those seaweeds. The highest production of total SCFAs, and acetic and propionic acids, was also noticed after the fermentation of *Gelidium*-extract [179].

A similar study was carried out by Kuda *et al.* [165], in order to verify the effectiveness of laminaran and alginate (LMW- and HMW-PS). They observed that both laminaran and alginate caused an increase in the production of total organic acids in the human feces in *in vitro* experiments. However, while the alginate induced an augmentation in the acetic acid levels, laminaran caused an increase in propionic, butyric and lactic acids. They also noticed that the release of putrefactive compounds decreased. During a concomitant experiment with rats, Kuda *et al.* noticed that only laminaran and LMW-alginate caused a positive effect on the organic acids in the cecal contents. Furthermore, as it happened with an *in vitro* experiment with human feces, putrefactive compounds decreased up to 60% in the cecum of the rats fed diets supplemented with laminaran and alginate from seaweeds. This might be due to the ability of these PS to inhibit the growth of putrefactive microorganisms. It is important to note that putrefactive compounds are related to the appearance of colon cancer and, thus, can be looked at as biomarkers for this disease [165]. Similar results were obtained by using a laminaran-oligosaccharide (DP 22) either *in vitro* or *in vivo*, which stimulated the growth of bifidobacteria as well. These researchers also found that a β-glucan from the microalga *Euglena gracilis* caused an increase in the stool bulk, but could not be fermented by human microflora [187] (Table 4).

Alginates were also proved to act as prebiotics in weaning piglets, by increasing the number of enterococci and improving bacterial diversity in the intestine [188]. Furthermore, an alginate-derived LMW-polymannuronate improved cecal microflora and lactic and acetic acids in the cecum of broiler chickens [189]. Despite the low number of individuals used in the trial, Terada and colleagues [60] already studied the prebiotic effects of alginate in humans two decades ago. They observed that alginate stimulated the growth of beneficial bacteria in the colon and inhibited harmful microorganisms, with the consequent (significant) decrease in putrefactive compounds in the feces. Alginate-fed individuals also presented higher levels of total SCFAs, and acetic and propionic acids.

In another study, Liu and co-workers [127] showed that the biomass from the microalgae *Chondrus crispus* also possesses prebiotic properties. These researchers fed rats a diet supplemented with *C. crispus* and verified that the animals’ microbiota was improved. The beneficial bacteria increased, as did the levels of the acetic, propionic and butyric SCFAs. An improvement of the histomorphology of the colon and an increase in the water-holding capacity of the feces were observed as well, as favorable effects provided by the biomass of the red seaweed. The immune status was also enhanced, as the levels of immunoglobulins A and G increased (Table 4).

In addition to seaweeds, some microalgae are also known to have prebiotic properties. For example, the biomass of *Arthrospira platensis* can promote the growth of beneficial bacteria, such as *Lactobacillus casei*, *Streptococcus thermophilus*, and *L. acidophilus* in special [87,180]. Furthermore, harmful pathogenic bacteria (*Proteus vulgaris*, *Bacillus subtilis* and *B. pumulis*, for example) were suppressed in an *in vitro* study [87]. When added to yogurt, the biomass from *Spirulina* promoted the growth of *L. acidophilus* and *Bifidobacteria* as well [181]. However, none of the effects on the production of SCFAs were determined in any of these studies. *Isochrysis galbana* is another marine microalga with high contents of both soluble and insoluble fibers, and it is promising as a prebiotic since the numbers of LAB increased in the feces of rats treated with *I. galbana* [182]. Nevertheless, some more studies are necessary in order to confirm the effectiveness of this microalga on the colon histomorphology and production of organic acids (Table 4).

There is also evidence that some algal PS are able to regulate the numbers of altered microbiota in mice [29,166]. Even so, these studies on the “selectivity of growth and/or activation of one species or a certain group of colon microorganisms” are scarce. Most of the research performed only evaluated the health benefits of the microflora in general.

For some of the benefits to be effective at the cell level, poly/oligosaccharides must present in specific glycan sequences that will match the respective receptors in the cells. This is related to the flexibility and rotation/torsion capacity of the molecules around their anomeric link. This flexibility is influenced by the substituents and the solvents, however [26,190].

The prebiotic properties provided by seaweeds and marine microalgae should not be restricted to their PS and lignin, but should rather be extended to monosaccharides, enzymes, polyunsaturated fatty acids (PUFAs), peptides, polyphenols, and alcohols, as it was demonstrated for similar compounds from other origins [35,191,192,193].

In the near future, the possibility of using PS from marine algae or oligosaccharides resultant thereof, through several degrading techniques, to modulate the microbiome, and, consequently, to prevent diseases is foreseen. These techniques may include new enzymes from bacteria and mollusks from marine origin.

## 4. Conclusions and Final Remarks

From a large number of *in vitro* and *in vivo* studies it becomes evident that the gut microbiota plays a much more important role upon the host’s well-being and health than it was previously realized, and the flora can be selectively modulated by a group of substances called prebiotics. Among these, a diverse group of polysaccharides can exert their action through a wide range of mechanisms, that include (i) selective fermentation; (ii) the gut pH; (iii) fecal bulking; (iv) the prevention of gut colonization by pathogens; (v) the control of putrefactive bacteria, therefore reducing the host’s exposure to toxic metabolites.

Algal oligo- and polysaccharides show effects on health similar to and sometimes more effective than other oligosaccharides from different sources. Their chemical structures include, at least partly, some of these oligosaccharides and some of the PS produced by seaweeds and marine microalgae that are not degraded by enzymes in the upper part of the GI tract. Therefore, algal PS present a great potential for emergent prebiotics to be used directly, in the case of microalgae, or as dried biomass or nutraceuticals, after extraction from the biomass or from the culture medium. They may be included in food and/or feed, or administered as pills, for example. The development of new enzyme technologies together with new enzymes from marine bacteria and mollusks will enable us to tune these PS and produce novel prebiotics.

## References

[1] Clemente J.C., Ursell L.K., Parfrey L.W., Knight R. (2012). The impact of the gut microbiota on human health: An integrative view. Cell.

[2] Institute of Medicine: Food and Nutrition Board (2005). Dietary Reference Intakes: Energy, Carbohydrates, Fiber, Fat, Fatty Acids, Cholesterol, Protein and Amino Acids.

[3] Turner N.D., Lupton J.R. (2011). Dietary fiber. Adv. Nutr..

[4] American Association of Cereal Chemists (2001). The definition of dietary fiber. Cereal Foods World.

[5] Codex Alimentarius Commission Guidelines for the use of nutrition claims: draft table of conditions for nutrient contents (Part B) provisions on dietary fibre. Proceedings of the Report of the 30th Session of the Codex Committee on Nutrition and Foods for Special Dietary Uses.

[6] Kraan S. (2012). Algal polysaccharides, novel applications and outlook. Carbohydrates—Comprehensive Studies on Glycobiology and Glycotechnology.

[7] Patarra R.F., Paiva L., Neto A.I., Lima E., Baptista J. (2011). Nutritional value of selected macroalgae. J. Appl. Phycol..

[8] Rupérez P., Saura-Calixto F. (2001). Dietary fiber and physicochemical properties of edible Spanish seaweeds. Eur. Food Res. Technol..

[9] Burtin P. (2003). Nutritional value of seaweeds. EJEAFChe.

[10] Dawczynski C., Schubert R., Jahrein G. (2007). Amino acids, fatty acids, and dietary fibre in edible seaweed products. Food Chem..

[11] Institut de Phytonutrition (2004). Functional, Health and Therapeutic Effects of Algae and Seaweed.

[12] Norziah M.H., Ching C.Y. (2000). Nutritional composition of edible seaweed *Gracilaria changgi*. Food Chem..

[13] Wong K.H., Cheung P.C.K. (2000). Nutritional evaluation of some red and green seaweeds, Part I—Proximate composition, amino acid profiles and some physico-chemical properties. Food Chem..

[14] Dvir I., Chayoth R., Sod-Moriah U., Shany S., Nyska A., Stark A.H., Madar Z., Arad S.M. (2000). Soluble polysaccharide and biomass of red microalga *Porphyridium* sp. alter intestinal morphology and reduce cholesterol in rats. Br. J. Nutr..

[15] Lahaye M., Jegou D. (1993). Chemical and physico-chemical characteristics of dietary fibers of *Ulva lactuca* (L.) Thuret and *Enteromorpha compressa* (L.) Grev. J. Appl. Phycol..

[16] Yoshie Y., Suzuki T., Shirai T., Hirano T. (1997). Analytical procedure and distribution of soluble and insoluble dietary fibers in seaweed foods. J. Tokyo Univ. Fish..

[17] McCance R.A., Widdowson E.M., Holland B. (1993). McCance and Widdowson’s Composition of Foods.

[18] MacArtain P., Gill C.I.R., Brooks M., Campbell R., Rowland I.R. (2007). Nutritional value of edible seaweeds. Nutr. Rev..

[19] McLachlan A.J., Morgan P.R., Howard-Williams C., McLachlan S.M., Bourn D. (1972). Aspects of the recovery of a saline African lake following a dry period. Arch. Hydrobiol..

[20] Pereira L., Pomin V.H. (2011). A review of the nutrient composition of selected edible seaweeds. Seaweed: Ecology, Nutrient Composition and Medicinal Uses.

[21] Praznik W., Loeppert R., Viernstein H., Haslberger A.G., Unger F.M., Ramawat K.G., Mérillon J.M. (2015). Dietary fiber and prebiotics. Polysaccharides: Bioactivity and Biotechnology.

[22] Meyer K.A., Kushi L.H., Jacobs D.R., Slavin J., Sellers T.A., Folsom A.R. (2002). Carbohydrates, dietary fiber, and incident type 2 diabetes in older women. Am. J. Clin. Nutr..

[23] Kasper H. (2000). Ernährungsmedizin und Diätetic.

[24] Baghurst P.A., Baghurst K.I., Record S.J. (1996). Dietary fiber, non-starch polysaccharides and resistant starch—A review. Food Aust..

[25] Potty V.H. (1996). Physico-chemical aspects, physiological functions, nutritional importance and technological significance of dietary fibers—A critical appraisal. J. Food Sci. Technol..

[26] Courtois J. (2009). Oligosaccharides from land plants and algae: Production and applications in therapeutics and biotechnology. Curr. Opin. Microbiol..

[27] Klarzynski V., Descamps V., Plesse B., Yvin J.C., Kloareg B., Fritig B. (2003). Sulfated fucan oligosaccharides elicit defense responses in tobacco and local systemic resistance against tobacco mosaic virus. Mol. Plant Microb. Interact..

[28] Chow J.T.N., Williamson D.A., Yates K.M., Goux W.J. (2005). Chemical characterization of the immunomodulating polysaccharide of *Aloe vera* L.. Carbohydr. Res..

[29] Zaporozhets T.S., Besednova N.N., Kusnetsova T.A., Zvyagintseva T.N., Makarenkova I.D., Kryzhanovsky S.P., Melnikov V.G. (2014). The prebiotic potential of polysaccharides and extracts of seaweeds. Russ. J. Mar. Bot..

[30] Kardos N., Luche J.L. (2001). Sonochemistry of carbohydrate compounds. Carbohydr. Res..

[31] Lii C.Y., Chen C.H., Yeh A.I., Lai V.M.F. (1999). Preliminary study on the degradation kinetics of agarose and carrageenans by ultrasound. Food Hydrocoll..

[32] Sun L., Wang C., Shi Q., Ma C. (2009). Preparation of different molecular weight polysaccharides from *Porphyridium cruentum* and their antioxidant activities. Int. J. Biol. Macromol..

[33] Zúñiga E.A., Matsuhiro B., Mejías E. (2006). Preparation of low-molecular weight fraction by free radical depolymerisation of the sulfated galactan from *Schizymenia binderi* (Gigartinales, Rhodophyta) and its anticoagulant activity. Carbohydr. Polym..

[34] Leal B.E.S. (2015). Obtenção de Oligossacarídeos Prebióticos a Partir da Hidrólise Fosfórica da Biomassa de Microalgas Utilizadas na Biomitigação de CO_2_ de Efluente Gasoso de Churrascaria. Master’s Thesis.

[35] Nardella A., Chaubet F., Boisson-Vidal C., Blondin C., Durand P., Jozefonvicz J. (1996). Anticoagulant low molecular weight fucans produced by radical process and ion exchange chromatography of high molecular weight fucans extracted from the brown seaweed *Ascophyllum nodosum*. Carbohydr. Res..

[36] Patel S., Goyal A. (2012). The current trends and future perspectives of prebiotics research: A review. 3 Biotech.

[37] Enoki T., Okuda S., Kudo Y., Takashima F., Sagawa H., Kato I. (2010). Oligosaccharides from agar inhibit pro-inflammatory mediator release by inducing heme oxygenase 1. Biosci. Biotechnol. Biochem..

[38] Claudia N.P., Matsuhiro B. (2008). Characterization of a fucoidan from *Lessonia vadose* (Phaeophyta) and its anticoagulant and elicitor properties. Int. J. Biol. Macromol..

[39] Zhou G., Sun Y.P., Xin H., Zhang Y., Li Z., Xu Z. (2004). *In vivo* antitumor and immunomodulation activities of different molecular weight lambda-carrageenans from *Chondrus ocellatus*. Pharmacol. Res..

[40] Sun L., Wang L., Zhou Y. (2012). Immunomodulation and antitumor activities of different molecular-weight polysaccharides from *Porphyridium cruentum*. Carbohydr. Res..

[41] Holck J., Hjernø K., Lorentzen A., Vigsnæs L.K., Hemmingsen L., Licht T.R., Mikkelsen J.D., Meyer A.S. (2011). Tailored enzymatic production of oligosaccharides from sugar beet pectin and evidence of differential effects of a single DP chain length difference on human faecal microbiota composition after *in vitro* fermentation. Proc. Biochem..

[42] Wang Z., Roberts A.B., Buffa A.J., Levison B.S., Zhu W., Org E., Gu X., Huang Y., Zamanian-Daryoush M., Culley M.K. (2015). Non-lethal inhibition of gut microbial trimethylamine production for the treatment of atherosclerosis. Cell.

[43] Rodrigues D., Rocha-Santos T.A.P., Gomes A.M., Goodfellow B.J., Freitas A.C. (2012). Lipolysis in probiotic and symbiotic cheese: The influence of probiotic bacteria, prebiotic compounds and ripening time on free fatty acid profiles. Food Chem..

[44] Raposo M.F.J., Morais A.M.M.B. (2015). Microalgae for the prevention of cardiovascular disease and stroke. Life Sci..

[45] Raposo M.F.J., Morais A.M.M.B., Morais R.M.S.C. (2015). Marine polysaccharides from algae with potential biomedical applications. Mar. Drugs.

[46] Raposo M.F.J., Morais A.M.M.B., Morais R.M.S.C., Ramawat K.G., Mérillon J.M. (2015). Polysaccharides from marine microalgae. Polysaccharides: Bioactivity and Biotechnology.

[47] Werman M.J., Sukenik A., Mokady S. (2003). Effects of the marine unicellular alga *Nannochloropsis* sp. to reduce the plasma and liver cholesterol levels in male rats fed on diets with cholesterol. Biosci. Biotechnol. Biochem..

[48] De Morais M.G., Vaz B.S., de Morais E.G., Costa J.A.V. (2015). Biologically active metabolites synthesized by microalgae. Biomed. Res. Int..

[49] Jimenez-Escrig A., Sanchez-Muniz F.J. (2000). Dietary fiber from edible seaweeds: Chemical, structure, physicochemical properties and effects on cholesterol metabolism. Nutr. Res..

[50] Pal A., Kamthania M.C., Kumar A. (2014). Bioactive compounds and properties of seaweeds—A review. OALib J..

[51] Lahaye M. (1991). Marine-algae as sources of fibers—Determination of soluble and insoluble dietary fiber contents in some sea vegetables. J. Sci. Food Agric..

[52] Lahaye M., Thibault J.F., Southgate D.A.T., Waldron K., Johnson I.T., Fenwick G.R. (1990). Chemical and physico-chemical properties of fibers from algae extraction by-products. Dietary Fibre: Chemical and Biological Aspects.

[53] Wood P.J., McCleary B.V., Prosky L. (2001). Cereal β-glucans: Structure, properties and health claims. Advanced Dietary Fibre Technology.

[54] Fleury N., Lahaye M. (1991). Chemical and physicochemical characterization of fibers from *Laminaria digitata* (Kombu Breton)—A physiological approach. J. Sci. Food Agric..

[55] Lahaye M., Roneau-Mouro C., Deniaud E., Buléon A. (2003). Solid-state ^13^C NMR spectroscopy studies of xylans in the cell wall of *Palmaria palmata* (L. Kuntze, Rhodophyta). Carbohydr. Res..

[56] Kimura Y., Watanabe K., Okuda H. (1996). Effects of soluble sodium alginate on cholesterol excretion and glucose tolerance in rats. J. Ethnopharmacol..

[57] Zee S. (1991). Body weight loss with the aid of alginic acid. Med. Arh..

[58] Vaugelade P., Hoebler C., Bernard F., Guillon F., Lahaye M., Duee P.H., Darcy-Vrillon B. (2000). Non-starch polysaccharides extracted from seaweed can modulate intestinal absorption of glucose and insulin response in the pig. Reprod. Nutr. Dev..

[59] Torsdottir I., Alpsten M., Holm G., Sandberg A.S., Tolli J. (1991). A small dose of soluble alginate-fiber affects postprandial glycemia and gastric-emptying in humans with diabetes. J. Nutr..

[60] Terada A., Hara H., Mitsuoka T. (1995). Effect of dietary alginate on the faecal microbiota and faecal metabolic activity in humans. Microbial Ecol. Health Dis..

[61] Wang Y., Han F., Hu B., Li J., Yu W. (2006). *In vivo* prebiotic properties of alginate oligosaccharides prepared through enzymatic hydrolysis of alginate. Nutr. Res..

[62] Morrissey J., Kraan S., Guiry M.D. (2001). A Guide to Commercially Important Seaweeds on the Irish Coast.

[63] Dumelod B.D., Ramirez R.P.B., Tiangson C.L.P., Barrios E.B., Panlasigui L.N. (1999). Carbohydrate availability of arroz caldo with lambda-carrageenan. Int. J. Food Sci. Nutr..

[64] Deville C., Damas J., Forget J., Dandrifosse G., Peulen O. (2004). Laminarin in the dietary fiber concept. J. Sci. Food Agric..

[65] Deville C., Gharbi M., Dandrifosse G., Peulen O. (2007). Study of the effects of laminarin, a polysaccharide from seaweed, on gut characteristics. J. Sci. Food Agric..

[66] Lahaye M., Michel C., Barry J.L. (1993). Chemical, physicochemical and *in vitro* fermentation characteristics of dietary-fibers from *Palmaria palmata* (L.) Kuntze. Food Chem..

[67] Li N., Zhang Q.B., Song J.M. (2005). Toxicological evaluation of fucoidan extracted from *Laminaria japonica* in Wistar rats. Food Chem. Toxicol..

[68] Burri S., Tato I., Nunes M.L., Morais R. (2011). Functional vegetable-based sausages for consumption by children. Food Nutr. Sci..

[69] Raposo M.F.J., Morais R.M.S.C., Morais A.M.M.B. (2013). Bioactivity and applications of sulphated polysaccharides from marine microalgae. Mar. Drugs.

[70] Southgate D.A.T., Southgate D.A.T., Waldron K., Johnson I.T., Fenwick G.R. (1990). Duetary fiber and health. Dietary Fiber: Chemical and Biological Aspects.

[71] Roberfroid M.B. (1993). Dietary fiber, inulin, and oligofructose: A review comparing their physiological effects. Crit. Rev. Food Sci. Nutr..

[72] Gibson G.R., Roberfroid M.B. (1995). Dietary modulation of the human colonic microbiota: Introducing the concept of prebiotics. J. Nutr..

[73] Gibson G.R., Probert H.M., van Loo J.A.E., Roberfroid M.B. (2004). Dietary modulation of human colonic microbiota: Updating the concept of prebiotics. Nutr. Res. Rev..

[74] Gibson G.R., Scott K.P., Rastall R.A., Tuohy K.M., Hotchkiss A., Dubert-Ferrandon A., Gareau M., Murphy E.F., Saulnier D., Loh G. (2010). Dietary prebiotics: Current status and new definition. Food Sci. Technol. Bull. Funct. Foods.

[75] Roberfroid M. (2007). Prebiotics: The concept revisited. J. Nutr..

[76] Binns N. (2013). Probiotics, Prebiotics and the Gut Microbiota.

[77] Fernandes J., Su W., Rahat-Rozenbloom S., Wolever T.M.S., Comelli E.M. (2014). Adiposity, gut microbiota, and faecal short chain fatty acids are linked in adult humans. Nutr. Diabetes.

[78] Vogt J.A., Wolever T.M.S. (2003). Faecal acetate is inversely related to acetate absorption from the human rectum and distal colon. J. Nutr..

[79] Cummings J.H., Gibson G.R., Macfarlane G.T. (1995). Short chain fatty acids. Human Colonic Bacteria: Role in Nutrition, Physiology and Pathology.

[80] Macfarlane G.T., Gibson G.R., Cummings J.H. (1992). Comparison of fermentation reactions in different regions of the colon. J. Appl. Bacteriol..

[81] Barcenilla A., Pride S.E., Martin J.C., Duncan S.H., Stewart C.S., Henderson C., Flint J.Y. (2000). Phylogenetic relationships of butyrate-producing bacteria from the human gut. Appl. Environ. Microbiol..

[82] Salminen S., Bouley C., Boutron-Ruault M.C., Cummings J.H., Franck A., Gibson G.R., Isolauri I., Moreau M.C., Roberfroid M., Rowland I.R. (1998). Functional food science and gastrointestinal function. Br. J. Nutr..

[83] Mykkanen H., Laiho K., Salminen S. (1998). Variations in faecal bacterial enzyme activities and associations with bowel function and diet in elderly subjects. J. Appl. Microbiol..

[84] Gibson G.R., Roberfroid M.B. (1999). Colonic Microbiota Nutrition and Health.

[85] Macfarlane G.T., McBain A.J., Gibson G.R., Roberfroid M.B. (1999). The human colonic microbiota. Colonic Microbiota Nutrition and Health.

[86] Van der Waaij D., Hanson L.A., Yolken R.H. (1999). Microbial ecology of the intestinal microflora: Influence of interactions with the host organism. Probiotics, Other Nutritional Factors, and Intestinal Microflora.

[87] Bhowmik D., Dubey J., Mehra S. (2009). Probiotic efficiency of *Spirulina platensis*-stimulating growth of lactic acid bacteria. World J. Dairy Food Sci..

[88] Conway P.L. (2001). Prebiotics and human health: The state-of-the-art and future perspectives. Food Nutr. Res..

[89] Furrie E., Macfarlane S., Kennedy A., Cummings J.H., Walsh S.V., O’Neil D.A., Macfarlane G.T. (2005). Synbiotic therapy (*Bifidobacterium longum*/Synergy 1) initiates resolution of inflammation in patients with active ulcerative colitis: A randomized controlled pilot trial. Gut.

[90] Lindsay J.O., Whelan K., Stagg A.J., Gobin P., Al-Hassi H.O., Rayment N., Kamm M.A., Knight S.C., Forbes A. (2006). Clinical, microbiological, and immunological effects of fructo-oligosaccharide in patients with Crohn’s disease. Gut.

[91] Silk D.B., Davis A., Vulevic J., Tzortzis G., Gibson G.R. (2009). Clinical trial: The effects of a trans-galactooligosaccharide prebiotic on faecal microbiota and symptoms in irritable bowel syndrome. Aliment. Pharmacol. Ther..

[92] Uchida M., Mogami O., Matsueda K. (2007). Characteristic of milk whey culture with *Propioni-bacterium freudenreichii* ET-3 and its application to the inflammatory bowel disease therapy. Inflammopharmacology.

[93] Kasubuchi M., Hasegawa S., Hiramatsu T., Ichimura A., Kimura I. (2015). Dietary gut microbial metabolites, short-chain fatty acids, and host metabolic regulation. Nutrients.

[94] Rafter J., Bennett M., Caderni G., Clune Y., Hughes R., Karlson P.C., Klinder A., O’Riordan M., O’Sullivan G.C., Pool.Zobel B. (2007). Dietary synbiotics reduce cancer risk factors in polypectomized and colon cancer patients. Am. J. Clin. Nutr..

[95] Rowland I.R., Rumney C.J., Coutts J.T., Lievense L.C. (1998). Effect of *Bifidobacterium longum* and inulin on gut bacterial metabolism and carcinogen-induced aberrant crypt focci in rats. Carcinogenesis.

[96] Demigne C., Jacobs H., Moundras C., Davicco M.J., Horcajada M.N., Berlanier A., Coxam V. (2008). Comparison of native or reformulated chicory fructans, or non-purified chicory, on rat cecal fermentation and mineral metabolism. Eur. L. Nutr..

[97] Abrams S.A., Griffin I.J., Hawthorne K.M., Liang L., Gunn S.K., Darlington G., Ellis K.J. (2005). A combination of prebiotic short- and long-chain inulin-type fructans enhance calcium absorption and bone mineralization in young adolescents. Am. J. Clin. Nutr..

[98] Cieślik E., Topolska K., Pisulewski P.M. (2009). Effect of inulin-type fructans on body weight gain and selected biochemical parameters at calcium hypoalimentation in rats. Pol. J. Food Nutr. Sci..

[99] Cummings J.H., Christie S., Cole T.J. (2001). A study of fructo-oligosaccharide in the prevention of travellers’ diarrhoea. Aliment. Pharmacol. Ther..

[100] Lewis S., Burmeister S., Brazier J. (2005). Effect of the prebiotic oligofructose on relapse of *Clostridium difficile*-associated diarrhea: A randomized, controlled study. Clin. Gastroenterol. Hepatol..

[101] Waligora-Dupriet A.J., Campeotto F., Nicolis I., Bonet A., Soulaines P., Dupont C., Butel M.J. (2007). Effect of oligofructose supplementation on gut microflora and well-being in young children attending a day care centre. Int. J. Food Microbiol..

[102] Sotnikova N., Antsiferova I., Malyshkina A. (2002). Cytokine network of eutopic and ectopic endometrium in women with adenomyosis. Am. J. Reprod. Immunol..

[103] Macfarlane G.T., Gibson S.R. (1991). The control and consequences of bacterial fermentation in the human colon. J. Appl. Bacteriol..

[104] Ley R.E., Bäckhed F., Turnbaugh P.J., Lozupone C.A., Knight R.D., Gordon J.I. (2005). Obesity alters gut microbial ecology. Proc. Natl. Acad. Sci. USA.

[105] Turnbaugh P.J., Ley R.E., Mahowald M.A., Magrini V., Mardis E.R., Gordon J.I. (2006). An obesity-associated gut microbiome with increased capacity for energy harvest. Nature.

[106] Ley R.E., Turnbaugh P.J., Klein S., Gordon J.I. (2006). Microbial ecology: Human gut microbes associated with obesity. Nature.

[107] Topping D.L., Clifton P.M. (2001). Short-chain fatty acids and human colonic function: Roles of resistant starch and non-starch polysaccharides. Physiol. Rev..

[108] Bird A.R., Brown I.L., Topping D.L. (2000). Starches, resistant starches, the gut microflora and human health. Curr. Issues Intest. Microbiol..

[109] Pluske J.R., Durmic Z., Pethick D.W., Mullan B.P., Hampson D.J. (1998). Confirmation of the role of rapidly fermentable carbohydrates in the expression of swine dysentery in pigs after experimental infection. J. Nutr..

[110] Topping D.L., Mock S., Trimble R.P., Illman R.J. (1988). Effects of varying the content and proportions of gum Arabic and cellulose on cecal volatile fatty acids in the rat. Nutr. Res..

[111] Baghurst K.I., Hope A.K., Down E.C. (1985). Dietary intake in a group of institutionalized elderly and the effects of a fibre supplementation program on nutrient intake and weight gain. Communit. Health Stud..

[112] Cherrington C.A., Hinton M., Pearson G.R., Chopra I. (1991). Short-chain organic acids at pH 5.0 kill *Escherichia coli* and *Salmonella* spp. without causing membrane perturbation. J. Appl. Bacteriol..

[113] Slavin J. (2013). Fiber and prebiotics: Mechanisms and health benefits. Nutrients.

[114] Brown I.L., Warhurst M., Arcot J., Playne M., Illman R.J., Topping D.L. (1997). Fecal numbers of bifidobacteria are high in pigs fed *Bifidobacterium longum* with a high amylose (amylomayze) starch than with a low amylomayze starch. J. Nutr..

[115] Campbell J.M., Fahey G.C., Wolf B.W. (1997). Selected indigestible oligosaccharides affect large bowel mass, cecal and fecal short-chain fatty acids, pH and microflora in rats. J. Nutr..

[116] Kleessen B., Sykura B., Zunft H.J., Blaut M. (1997). Effects of inulin and lactose on fecal microflora, microbial activity, and bowel habit in elderly constipated persons. Am. J. Clin. Nutr..

[117] Ohkusa T., Ozaki Y., Sato C., Mikuni K., Ikeda H. (1995). Long-term ingestion of lactosucrose increases *Bifidobacterium* sp. in human fecal flora. Digestion.

[118] Dimitroglou A., Davies S.J., Sweetman J., Divanach P., Chatzifotis S. (2010). Dietary supplementation of mannan oligosaccharide on white sea bream (*Diplodus sargus* L.) larvae: Effects on development, gut morphology and salinity tolerance. Aquacult. Res..

[119] Marinho M.C., Pinho M.A., Mascarenhas R.D., Silva F.C., Lordelo M.M., Cunha L.F., Freire J.P.B. (2007). Effect of prebiotic or probiotic supplementation and ileo rectal anastomosis on intestinal morphology of weaned piglets. Livest. Sci..

[120] Pérez-Conesa D., López G., Ros G. (2007). Effects of probiotic, prebiotic and symbiotic follow-up infant formulas on large intestine morphology and bone mineralisation in rats. J. Sci. Food Agric..

[121] Mourão J.L., Pinheiro V., Alves A., Guedes C.M., Pinto L., Saavedra M.J., Spring P., Kocher A. (2006). Effect of mannan oligosaccharides on the performance, intestinal morphology and cecal fermentation of fattening rabbits. Anim. Feed Sci. Technol..

[122] Spencer J.D., Touchete K.J., Liu H., Alle G.L., Newcom M.D., Kerley M.S., Pace L.W. (1997). Effect of spray-dried plasma and fructooligosaccharide on nursery performance and small intestinal morphology on weaned pigs. J. Anim. Sci..

[123] Ohta A., Osakabe N., Yamada K., Saito Y., Hidaka H. (1993). Effects of fructooligosaccharides and other saccharides on Ca, Mg and P absorption in rats. J. Jpn. Soc. Nutr. Food Sci..

[124] Chonan O., Watanuki M. (1995). Effect of galactooligosaccharides on calcium absorption in rats. J. Nutr. Sci. Vitaminol. (Tokyo).

[125] Salgado P., Martins J.M., Carvalho F., Abreu M., Freire J.P.B., Toullec R., Lalles J.P., Bento O. (2002). Component digestibility of lupin (*Lupinus angustifolius*) and pea (*Pisum sativum*) seeds and effects on the small intestine and body organs in anastomosed and intact growing pigs. Anim. Feed Sci. Technol..

[126] Fuller M.F., Verstegen M.W.A., Huisman J., den Hartog L.A. (1991). Methodologies of the measurement of digestion. Proceedings of the 5th International Symposium on Digestive Physiology in Pigs.

[127] Liu J., Kandasamy S., Zhang J., Kirby C.W., Karakach T., Hafting J., Crichley A.T., Evans F., Prithiviraj B. (2015). Prebiotic effects of diet supplemented with the cultivated red seaweed *Chondrus crispus* or with fructo-oligosaccharide on host immunity, colonic microbiota and gut microbial metabolites. BMC Complement. Altern. Med..

[128] Resta-Lenert S., Barrett K.E. (2003). Live probiotics protect intestinal epithelial cells from the effects of infection with enteroinvasive *Escherichia coli* (EIEC). Gut.

[129] Brown A.J., Goldworthy S.M., Barnes A.A., Eilert M.M., Tcheang I., Daniels D., Muir A.I., Wigglesworth M.J., Kinghorn I., Fraser N.J. (2003). The orphan G protein-coupled receptors GPR41 and GPR43 are activated by propionate and other short chain carboxylic acids. J. Biol. Chem..

[130] Nilsson N.E., Kotarsky K., Owman C., Olde B. (2003). Identification of a free fatty acid receptor, FFAR2R, expressed on leukocytes and activated by short-chain fatty acids. Biochem. Biophys. Res. Commun..

[131] Kimura I., Inoue D., Hirano K., Tsujimoto G. (2014). The SCFA receptor GPR43 and energy metabolism. Front. Endocrinol..

[132] Du H., van der A D.L., Boshuizen H.C., Forouhi N.G., Wareham N.J., Halkjaer J., TjØnnland A., Overvad K., Jakobsen M.U., Boeing H. (2010). Dietary fiber and subsequent changes in body weight and waist circumference in European men and women. Am. J. Clin. Nutr..

[133] Anderson J.W., Baird P., Davis R.H., Ferreri S., Knudtson M., Koraym A., Waters V., Williams C.L. (2009). Health benefits of dietary fibre. Nutr. Rev..

[134] Liu S., Willet W.C., Manson J.E., Hu F.B., Rosner B., Colditz G. (2003). Relation between changes in intakes of dietary fibre and grain products and changes in weight and development of obesity among middle-aged women. Am. J. Clin. Nutr..

[135] Freeland K.R., Wolever T.M. (2010). Acute effects of intravenous and rectal acetate on glucagon-like peptide-1, peptide YY, ghrelin, adiponectin and tumour necrosis factor-alpha. Br. J. Nutr..

[136] Tarini J., Wolever T.M. (2010). The fermentable fibre inulin increases postprandial serum short-chain fatty acids and reduces free-fatty acids and ghrelin in healthy subjects. Appl. Physiol. Nutr. Metab..

[137] Atarashi K., Tanoue T., Shima T., Imaoka A., Kuwahara T., Momose Y., Cheng G., Yamasaki S., Saito T., Ohba Y. (2011). Induction of colonic regulatory T cells by indigenous *Clostridium* species. Science.

[138] Round J.L., Mazmanian S.K. (2010). Inducible Foxp3+ regulatory T-cell development by a commensal bacterium of the intestinal microbiota. Proc. Natl. Acad. Sci. USA.

[139] HØverstad T., Midtvedt T. (1986). Short-chain fatty acids in germfree mice and rats. J. Nutr..

[140] Smith P.M., Howitt M.R., Panikov N., Michaud M., Gallini C.A., Bohlooly-Y M., Glockman J.N., Garrett W.S. (2013). The microbial metabolites, short chain fatty acids, regulate colonic Treg cell homeostasis. Science.

[141] Cummings J.H., Pomare E.W., Branch W.J., Naylor C.P., Macfarlane G.T. (1987). Short chain fatty acids in human large intestine, portal, hepatic, and venous blood. Gut.

[142] Vinolo M.A.R., Rodrigues H.G., Nachbar R.T., Curi R. (2011). Regulation of inflammation by short chain fatty acids. Nutrients.

[143] Waldecker M., Kautenburger T., Daumann H., Busch C., Schrenk D. (2008). Inhibition of histone-deacetylase activity by short-chain fatty acids and some polyphenol metabolites formed in the colon. J. Nutr. Biochem..

[144] Hinnebusch B.F., Meng S., Wu J.T., Archer S.Y., Hodin R.A. (2002). The effects of short-chain fatty acids on human colon cancer cell-phenotype are associated with histone hyperacetylation. J. Nutr..

[145] Sealy L., Chalkley R. (1978). The effect of sodium butyrate on histone modification. Cell.

[146] Morgan D.O. (1995). Principles of CDK regulation. Nature.

[147] Newmark H.L., Young C.W. (1995). Butyrate and phenylacetate as differentiating agents: Practical problems and opportunities. J. Cell Biochem..

[148] Boffa L.C., Lupton J.R., Mariani M.R. (1992). Modulation of colonic epithelial cell proliferation, histone acetylation, and luminal short chain fatty acids by variation of dietary fiber in rats. Cancer Res..

[149] Medina V., Young G.P., Edmonds B., James R., Appleton S., Zalewski D.P. (1997). Induction of caspase-3 protease activity and apoptosis by butyrate and trichostatin a (inhibitors of histone deacetylase): Dependence on protein synthesis and synergy with a mitochondrial/cytochrome C-dependent pathway. Cancer Res..

[150] Grunstein M. (1997). Histone acetylation in chromatin structure and transcription. Nature.

[151] Meijer K., de Vos P., Priebe M.G. (2010). Butyrate and other short-chain fatty acids as modulators of immunity: What relevance for health?. Curr. Opin. Nutr. Metab. Care.

[152] Cavaglieri C.R., Nishiyama A., Fernandes L.C., Curi R., Miles E.A., Calder P.C. (2003). Differential effects of short-chain fatty acids on proliferation and production of pro- and anti-inflammatory cytokines by cultured lymphocytes. Life Sci..

[153] Doty M.S., Caddy J.F., Santelices B. (1987). Case Studies of Seven Commercial Seaweed Resources. FAO Fisheries Technical Paper-281, Food and Agriculture Organization of the United Nations.

[154] Agrimer, Algues Marines. http://www.agrimer.com/en/algues/2-brown/7-ascophyllum-nodosum.html.

[155] Agrimer, Algues Marines. http://www.agrimer.com/en/algues/2-brown/11-fucus-vesiculosus.html.

[156] Ramsden L., Tomasik P. (2004). Plant and algal gums and mucilages. Chemical and Functional Properties of Food Saccharides.

[157] Wu J.H., Xu C., Shan C.Y., Tan R.X. (2006). Antioxidant properties and PC12 cell protective effects of APS-1, a polysaccharide from *Aloe vera* var. *chinensis*. Life Sci..

[158] Hu B., Gong Q.N., Wang Y., Ma Y., Li J., Yu W. (2006). Prebiotic effects of neoagaro-oligosaccharides prepared by enzymatic hydrolysis of agarose. Anaerobe.

[159] Muraoka T., Ishihara K., Oyamada C., Kunitake H., Hirayama I., Kimura T. (2008). Fermentation properties of low-quality red alga Susabinori *Porphyra yezoensis* by intestinal bacteria. Biosci. Biotechnol. Biochem..

[160] Ray B., Lahaye M. (1995). Cell-wall polysaccharides from the marine green alga *Ulva rigida* (Ulvales, Chlorophyta); chemical structure of ulvan. Carbohydr. Res..

[161] Akiyama H., Endo T., Nakakita R., Murata K., Yonemoto Y., Okayama K. (1992). Effect of depolymerized alginates on the growth of bifidobacteria. Biosci. Biotechnol. Biochem..

[162] Pokusaeva K., Fitzgerald G., Sinderen D. (2011). Carbohydrate metabolism in bifidobacteria. Genes Nutr..

[163] Osipov G.A., Parfenov A.I., Verkhovtseva N.V., Ruchkina I.N., Kurchavov V.A., Boiko N.B., Rogatina E.L. (2003). The clinical significance of the study of microorganisms on the intestinal mucous membrane by cultural-biochemical and chromatography-mass spectrometry methods. Exp. Klin. Gastroenterol..

[164] Kusaikin M.I. (2003). O-glycosyl hydrolases of marine invertebrates. Properties and specifics of fucoidanases, sulfatases, and 1-3-beta-d-glucanases. Ph.D. Thesis.

[165] Kuda T., Yano T., Matsuda N., Nishizawa M. (2005). Inhibitory effects of laminaran and low molecular alginate against the putrefactive compounds produced by intestinal microflora *in vitro* and in rats. Food Chem..

[166] Kusnetsova T.A., Zaporozhets T.S., Makarenkova I.D., Besednova N.N., Timchenko N.F., Zvyagintseva T.N., Shevchenko N.M., Mandrakova N.V., Melnikov V.G. (2012). The prebiotic potential of polysaccharides from the brown alga *Fucus evanescens* and significance for the clinical use. Pac. Med. J..

[167] Gibson G.R., Roberfroid M.B. (2008). Handbook of Prebiotics.

[168] Koneva E.L. (2009). Substantiation and development of technologies for alginate-containing functional products. Ph.D. Thesis.

[169] Yamada Y., Miyoshi T., Tanada S., Imaki M. (1991). Digestibility and energy availability of wakame (*Undaria pinnatifida*) seaweed in Japanese. Nippon Eiseigaku Zasshi.

[170] Michel C., Lahaye M., Bonnet C., Mabeau S., Barry J.L. (1996). *In vitro* fermentation by human faecal bacteria of total and purified dietary fibers from brown seaweeds. Br. J. Nutr..

[171] O’Doherty J.V., Dillon S., Figat S., Callan J.J., Sweeney T. (2010). The effects of lactose inclusion and seaweed extract derived from *Laminaria* spp. on performance, digestibility of diet components and microbial populations in newly weaned pigs. Anim. Feed Sci. Technol..

[172] McDonnel P., Figat S., O’Doherty J.V. (2010). The effect of dietary laminarin and fucoidan in the diet of the weanling piglet on performance, selected fecal microbial populations and volatile fatty acid concentrations. Animal.

[173] Gudiel-Urbano M., Goni I. (2002). Effect of edible seaweeds (*Undaria pinnatifida* and *Porphyra tenera*) on the metabolic activities of intestinal microflora in rats. Nutr. Res..

[174] Dierick N., Ovyn A., de Smet S. (2009). Effect of feeding intact brown seaweed *Ascophyllum nodosum* on some digestive parameters and on iodine content in edible tissues in pigs. J. Sci. Food Agric..

[175] Reilly P., O’Doherty J.V., Pierce K.M. (2008). The effects of seaweed extract inclusion on gut morphology, selected intestinal microbiota, nutrient digestibility, volatile fatty acids concentration and the immune status of the weaned pig. Animal.

[176] Lynch M.B., Sweeney T., Callan J.J., O’Sullivan J.T., O’Doherty J.V. (2010). The effects of dietary *Laminaria*-derived laminarin and fucoidan on nutrient digestibility, nitrogen utilization, intestinal microflora and volatile fatty acid concentration in pigs. J. Sci. Food Agric..

[177] Enoki T., Sagawa H., Tominaga T., Nishiyama E., Koyama N., Sakai T., Kato I. (2003). Drugs, Foods or Drinks with the Use of Algae-Derived Physiologically Active Substances. U.S. Patent.

[178] Fernandez L.I., Valiente O.G., Mainardi V., Bello J.L., Velez H., Rosado A. (1989). Isolation and characterization of an antitumor active agar-type polysaccharide of *Gracilaria dominguensis*. Carbohydr. Res..

[179] Ramnani P., Chitarrari R., Tuohy K., Grant J., Hotchkiss S., Philp K., Campbell R., Gill C., Rowland I. (2012). *In vitro* fermentation and prebiotic potential of novel low molecular weight polysaccharides derived from agar and alginate seaweeds. Anaerobe.

[180] Parada J.L., de Caire G.Z., de Mule M.C.Z., de Cano M.M.S. (1998). Lactic acid bacteria growth promoters from *Spirulina platensis*. Int. J. Food Microbiol..

[181] Beheshtipour H., Mortazavian A.M., Haratian P., Darani K.K. (2012). Effects of *Chlorella vulgaris* and *Arthrospira platensis* addition on viability of probiotic bacteria in yogurt and its biochemical properties. Eur. J. Food Res. Technol..

[182] Nuño K., Villaruel-López A., Puella-Pérez A.M., Romero-Velarde E., Puela-Mora A.G., Ascencio F. (2013). Effects of the marine microalgae *Isochrysis galbana* and *Nannochloropsis oculata* in diabetic rats. J. Funct. Foods.

[183] Tokita Y., Nakajima K., Mochida H., Iha M., Nagamine T. (2010). Development of a fucoidan-specific antibody and measurement of fucoidan in serum and urine by sandwich ELISA. Biosci. Biotechnol. Biochem..

[184] Irhimeh M.R., Fitton J.H., Lowenthal R.M., Kongtawelert P. (2005). A quantitative method to detect fucoidan in human plasma using a novel antibody. Methods Find. Exp. Clin. Pharmacol..

[185] Kuda T., Goto H., Yokoyama M., Fujii T. (1998). Fermentable dietary fiber in dried products of brown algae and their effects on caecal microflora and levels of plasma lipids in rats. Fish. Sci..

[186] Sugano Y., Terada I., Arita M., Noma M., Matsumoto T. (1993). Purification and characterization of a new agarase from a marine bacterium, *Vibrio* sp. strain JT0107. Appl. Environ. Microbiol..

[187] Kuda T., Enomoto T., Yano T. (2009). Effects of two storage β-1,3-glucans, laminaran from *Eicenia bicyclis* and paramylon from *Euglena gracilis*, on cecal environment and plasma lipid levels in rats. J. Funct. Foods.

[188] Janczyk P., Pieper R., Smidt H., Souffrant W.B. (2010). Effect of alginate and inulin on intestinal microbial ecology of weanling pigs reared under different husbandry conditions. FEMS Microbiol. Ecol..

[189] Zhu W., Li D., Wang J., Wu H., Xia X., Bi W., Guan H., Zhang L. (2015). Effects of polymannuronate on performance, antioxidant capacity, immune status, cecal microflora, and volatile fatty acids in broiler chickens. Poult. Sci..

[190] Gawronski M., Conrad H., Springer T., Stahmann K.P. (1996). Conformational changes of the polysaccharide Cinerean in aqueous solution. Macromolecules.

[191] Yangilar F. (2013). The application of dietary fibre in food industry: Structural features, effects on health and definition, obtaining and analysis of dietary fibre: A review. J. Food Nutr. Res..

[192] Raposo M.F.J., Morais R.M.S.C., Morais A.M.M.B. (2013). Health applications of bioactive compounds from marine microalgae. Life Sci..

[193] Shenderov B.A. (2001). Medical microbial ecology and functional foods. Prebiotics and Functional Foods.

